# Supplementary material for: The scaling laws of edge vs. bulk interlayer conduction in mesoscale twisted graphitic interfaces
Source: Nat Commun. 2020 Sep 21;11:4746. doi: 10.1038/s41467-020-18597-0 (PMC7506013; doi:10.1038/s41467-020-18597-0)
Supplement: Supplementary file 1 — Supplementary Information [file 41467_2020_18597_MOESM1_ESM.pdf]

The scaling laws of edge vs. bulk interlayer conduction in mesoscale twisted graphitic interfaces

Supporting Information

Debopriya Dutta,[1‡] Annabelle Oz,[2‡], Oded Hod,[2] and Elad Koren[1*]

[1]*Faculty of Materials Science and Engineering and the Russell Berrie Nanotechnology Institute, Technion – Israel Institute of Technology, 3200003 Haifa, Israel.*

[2]*Department of Physical Chemistry, School of Chemistry, The Raymond and Beverly Sackler Faculty of Exact Sciences and The Sackler Center for Computational Molecular and Materials Science, Tel Aviv University, Tel Aviv, IL 6997801.*

The supporting information contains the following items:

1. Lateral Force Measurements
2. Fitting Procedure to Extract Interfacial Bulk and Edge Resistance
3. Experimental Data of Current vs. Voltage on a Single Graphitic Contact
4. Electronic Transport Calculations
5. Validity Test for the Value of the Broadening Factors
6. Convergence Test with respect to the Number of Modeled Layers
7. Illustration of the Wave Function of Eight Eigenstates Within the Fermi Window for Circular Junctions

‡ Equal authors contribution

* email: eladk@technion.ac.il

1. Lateral Force Measurements

The lateral shear force was measured during the sliding process in order to verify that sliding is performed under superlubric conditions, thus ensuring the existence of an angular mismatch at the bilayer graphene interface[1]. The shear force was evaluated using the relation $F = 2\sigma r$, applicable for small shear distances[1], where r is the mesa radius and $\sigma = 0.227$ [J·m^{-2}] is the adhesion energy of graphite[1]. Supplementary Figure 1 shows the lateral displacement (top panel), the measured lateral force (middle panel), and current (bottom panel) as function of time. The lateral force oscillations as well as the average friction (calculated as the difference between the force averages along the trace and retrace measurements) are smaller than 10 nN indicating that sliding is performed under superlubric conditions and that there is a rotational mismatch of $5° - 15°$ between the bottom and top graphene layers[1].

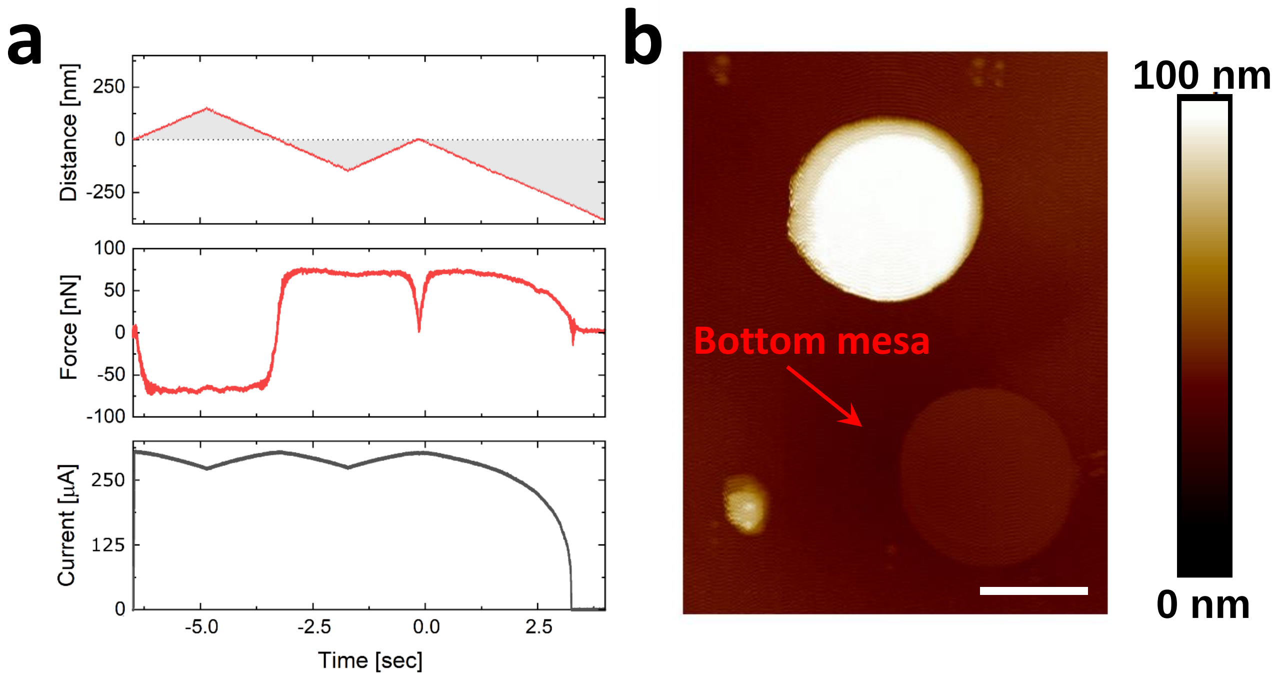

Supplementary Figure 1: Electromechanical manipulation of graphitic contacts. (a) lateral displacement (top panel), measured lateral force (middle panel), and current (bottom panel) as a function of time. The measured structure is 300 nm in diameter, the applied voltage is 1 V, and the tip velocity is 100 nm·sec^{-1}. **(b)** AFM image of a fully sheared graphitic mesa. The exposed bottom mesa consists a single crystalline graphitic surface with an arithmetic average surface roughness, $R_a = 0.108\ nm$.

We note that when shear forces are applied to bare graphitic surfaces, peeling and rippling effects may appear. Nevertheless, the situation in our experiments is quite different, as the sliding interface is buried deep inside the pillar and is supported by two thick graphitic slabs. This induces very strict constraints on the out-of-plane motion of the carbon atoms. Furthermore, as discussed above, our sliding interfaces are incommensurate, thus the shear forces are expected to be very small and hence rippling and buckling are highly unlikely to occur. One may think that some rippling may occur at the exposed surfaces during the sliding process. Nevertheless, we do not see any evidence for such rippling in the frictional behavior of the system, that demonstrates power law scaling of the friction forces with the contact area with an exponent of 0.3 as expected for incommensurate flat circular contacts[1,2]. This is also supported by the fact that there is no experimental evidence of wear in our contacts (Supplementary Figure 1b). Furthermore, the bottom panel of Fig. 1A (see main text) shows that the measured current is symmetric with respect to the fully-eclipsed configuration. Namely, for a given absolute shift value the current is the same when the top mesa shifts towards the center or away from it. If, in the former case, puckering of the lower surface would occur in front of the sliding surface[3], the current would not be symmetric. Another experimental support for these claims comes from the fact that all current vs. shift distance scaling laws derived in this paper rely on the circular geometry of the interface. Random rippling and buckling effect would not obey such straight-forward scaling laws with the shift distance. Based on all the above, we may exclude surface rippling effects and rely on the rigid sliding interface model.

2. Fitting Procedure to Extract Interfacial Bulk and Edge Resistance

A numerical fitting procedure was employed to obtain the current vs. sliding distance profiles, $I(x)$, and to extract the corresponding resistance of the sheared interface, R_{int}, and its Edge (*E*) and Bulk (*B*) contributions. $I(x)$ was calculated based on the equivalent electrical circuit depicted in Fig. 1d of the main text, i.e. $I(x) = \frac{V_{applied}}{\{2\times R_{Gr}+R_{sys}+R_{int}\}}$, where $R_{int} = \left[\left(R_{int}^{Bulk}\right)^{-1} + \left(R_{int}^{Edge}\right)^{-1}\right]^{-1}$. The bulk and edge interfacial resistances are related to the lateral sliding distance, x, via $R_{int}^{Bulk} = \frac{\rho^{Bulk}}{S^{Bulk}(x)}$ and $R_{int}^{Edge} = \frac{\rho^{Edge}}{L^{Edge}(x)}$, where $S^{Bulk}(x)$ and $L^{Edge}(x)$ are given by Eqs. 1 and 2 of the main text, respectively, and the corresponding resistivities ρ_{Bulk} and ρ_{Edge} serve as fitting parameters. The values of R_{Gr} and R_{sys} (considered herein to be constant throughout the sliding) are obtain from $I(x = 0)$. The interface voltage (in Fig. 2d) is extracted via: $V_{int} = I \cdot R_{int}$. The quality of the numerical fit is assessed based on a χ^2 test, where the average sum of the differences between calculated and measured currents, $\chi^2 = \sum_{i=1}^{n} \frac{(I_{calc}-I_{meas})^2}{n}$, is minimized. Here, I_{calc} and I_{meas} are the calculated and measured currents, respectively and n is the number of data points. Typical results for two current vs. slides curves measured under different bias voltages and their χ^2 diagram are shown in Supplementary Figure 2.

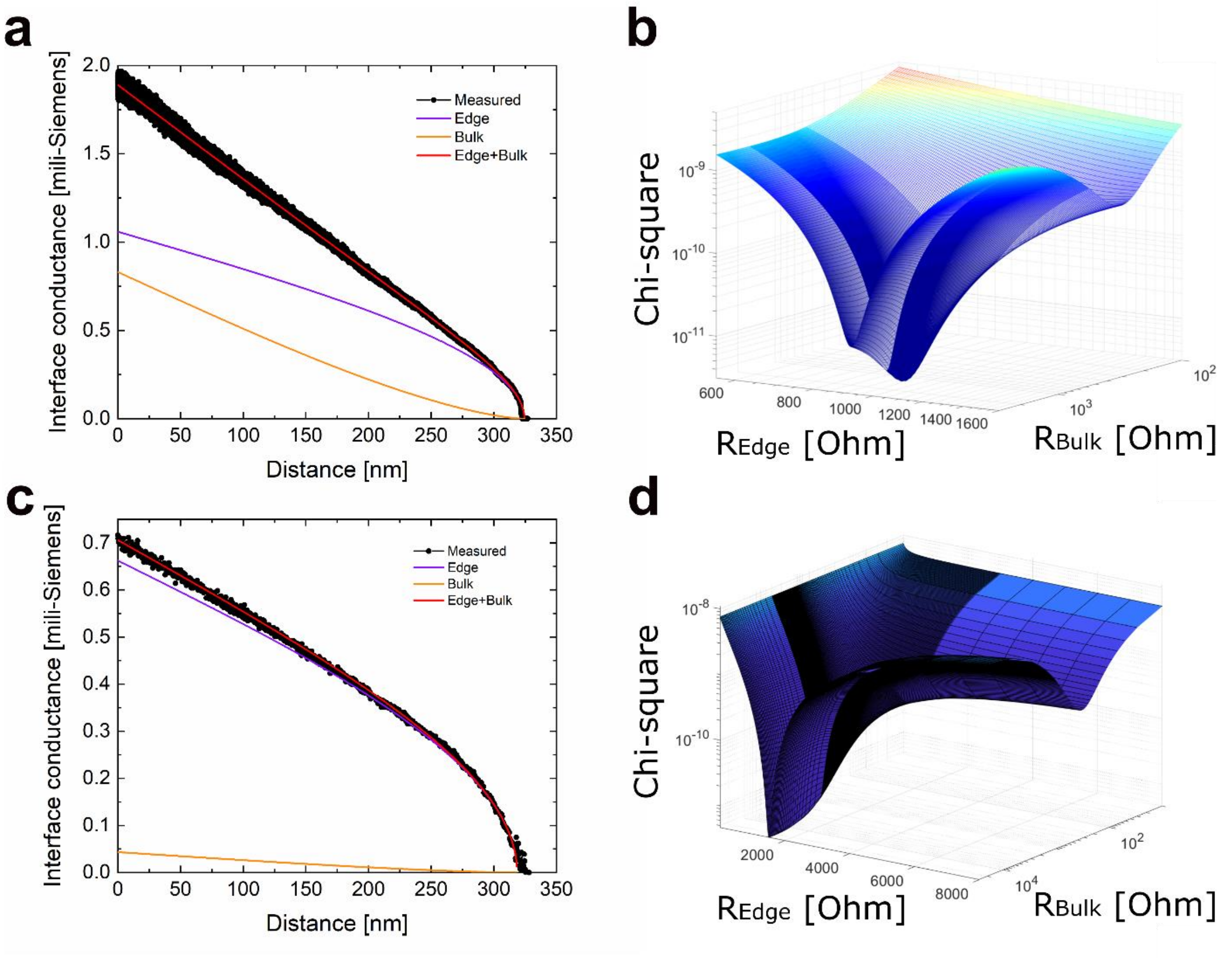

Supplementary Figure 2: Experimental determination of edge and bulk interlayer transport contributions in a graphitic contact. (a) Measured (black) and fitted (red) interface conductivities for an applied voltage of 1 V. The total calculated conductivity (red) is the sum of bulk (orange) and edge (purple) interface conductivities. **(b)** χ^2 for different edge and bulk interface resistances considered in the numerical fitting procedure. The χ^2 minimum corresponds to the optimally fitted interface edge and bulk resistance contributions. **(c)** Measured (black) and fitted (red) interface conductivities for an applied voltage of 0.5 V; and **(d)** its corresponding χ^2 diagram.

3. Experimental Data of Current vs. Voltage on a Single Graphitic Contact

In the main text, we presented surface plots of the current vs. voltage and interfacial shearing distance (Fig. 2a). To construct these diagrams using the same contact, we performed the experiment by shearing the interface in steps of 5 nm and measuring a full current-voltage profile for each shift position. Supplementary Figure 3a shows the original experimental data of the measured current vs. time taken from the oscilloscope. Supplementary Figure 3b provides a zoom-in on a region of two consecutive 5 nm sliding steps and voltage sweeps (between -1 V and 1 V) cycles. The measured current vs. voltage curves for each shearing position are then taken to construct the surface plots shown in Fig. 2a of the main text.

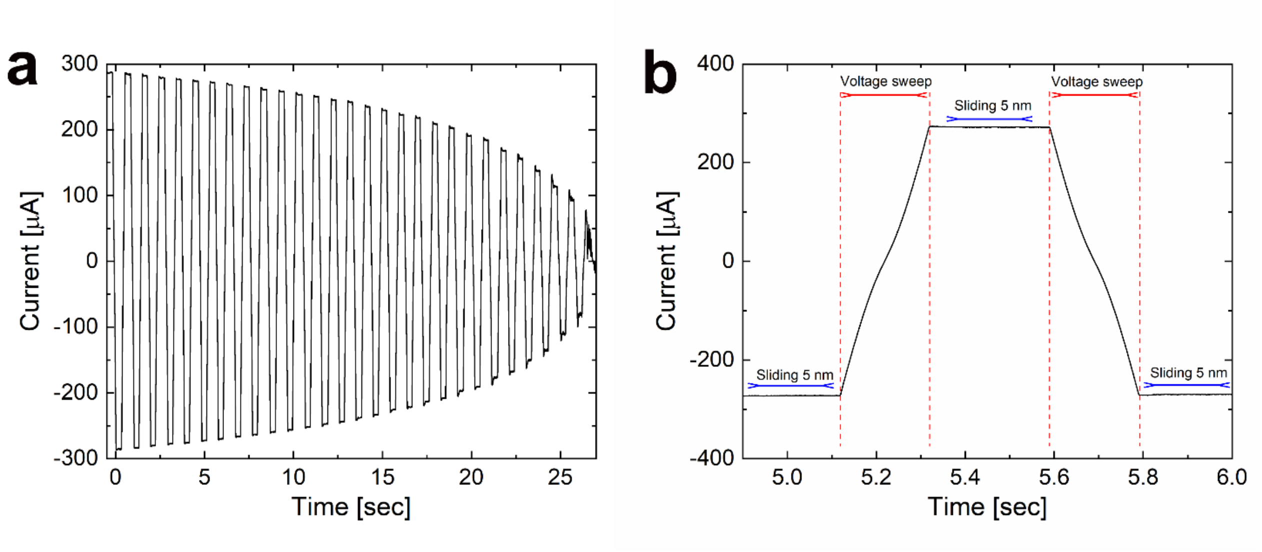

Supplementary Figure 3: Reconstruction of current-voltage profiles as a function of interfacial mesas overlap. (a) A typical current vs. time plot measured by shearing the interface in steps of 5 nm and measuring a full current-voltage profile for each shift position, where the voltage is swept from -1 V to 1 V. The contact diameter is 300 nm and the tip velocity during the sliding steps is 100 nm·sec^{-1}. **(b)** Zoom-in on a region of two consecutive sliding and voltage sweeps cycles.

4. Electronic Transport Calculations

To evaluate the interlayer transport behavior of the graphene junction (modeled herein as a bilayer graphene system composed of two finite hexagonal or circular graphene flakes) as a function of the layers relative position and bias voltage we adopt the approach presented in Ref. 4. The current-voltage characteristics are evaluated via the Landauer scattering formalism[5] under which the current,

$$I = \frac{2e}{h} \int \mathrm{d}E [f_T(E) - f_B(E)] T(E) \tag{1}$$

is related to the transmittance probability through the system, $T(E)$. In Supplementary Eq. (1), e is the electron charge, h is Plank's constant and

$$f_{T/B}(E) \equiv f_{T/B}(E; \mu_{T/B}, \beta_{T/B}) = \left[1 + e^{\beta_{T/B}(E - \mu_{T/B})}\right]^{-1} \tag{2}$$

is the equilibrium electronic Fermi-Dirac distribution of the top/bottom lead. Here, $\beta_{T/B} = (k_B T_{T/B})^{-1}$ are the inverse electronic thermal energies of the top (T) and bottom (B) leads, $T_{T/B}$ are the corresponding electronic temperatures, and the chemical potentials of the leads are assumed to evenly split the bias voltage, V_b, around the ground state Fermi energy of the entire finite model system, E_F, such that $\mu_{T/B} = E_F \pm 0.5eV_b$. The factor of two appearing in Supplementary Eq. (1) accounts for spin degeneracy. Note that, in the present treatment, we neglect the self-consistent effect of the electric field drop across the junction due to the externally applied bias voltage on the transmittance probability. This approximation can be partly justified by the semi-metallic nature of the graphene layers that may lead to screening effects, where most of the potential drop would occur at the lead/system interface.

The transmittance probability $T(E)$ appearing in Supplementary Eq. (1), is calculated using the non-equilibrium Green's function technique for elastic electronic transport:

$$T(E) = \mathrm{Tr}[\boldsymbol{G}_d^r(E) \boldsymbol{\Gamma}_B(E) \boldsymbol{G}_d^a(E) \boldsymbol{\Gamma}_T(E)]. \tag{3}$$

Here, $\boldsymbol{G}_d^{r/a}(E)$ are the retarded (r) and advanced (a) Green's function matrix representations of the junction and $\boldsymbol{\Gamma}_{T/B}(E)$ are the top and bottom broadening matrices. The formers are given by:

$$\boldsymbol{G}_d^r(E) = [E\boldsymbol{I} - \boldsymbol{h}_d - \boldsymbol{\Sigma}_B^r(E) - \boldsymbol{\Sigma}_T^r(E)]^{-1}, \tag{4}$$

and $\boldsymbol{G}_d^a(E) = [\boldsymbol{G}_d^r(E)]^\dagger$, where $\boldsymbol{I}$ is a unit matrix of dimensions of the system and $\boldsymbol{h}_d$ is the matrix representation of the device's Hamiltonian given by the following block form:

$$\boldsymbol{h}_d = \begin{pmatrix} \boldsymbol{h}_T & \boldsymbol{v}_{TB} \\ \boldsymbol{v}_{BT} & \boldsymbol{h}_B \end{pmatrix}, \tag{5}$$

where $\boldsymbol{h}_{T/B}$ are the Hamiltonian blocks of the top and bottom flakes given in the tight-binding atomic basis representation and $\boldsymbol{v}_{BT} = \boldsymbol{v}_{TB}^{\dagger}$ are their mutual coupling matrices. Note that in the present treatment the Hermitian matrix $\boldsymbol{h}_d$ is taken to be real-valued and is therefore symmetric. The leads self-energies are approximated as energy independent (wide band approximation) diagonal matrices providing the same lifetime, γ^{-1}, to each atomic site in the top and bottom sections:

$$\boldsymbol{\Sigma}_T^r = i\hbar\gamma \begin{pmatrix} \boldsymbol{I}_T & \boldsymbol{0} \\ \boldsymbol{0} & \boldsymbol{0} \end{pmatrix}, \boldsymbol{\Sigma}_B^r = i\hbar\gamma \begin{pmatrix} \boldsymbol{0} & \boldsymbol{0} \\ \boldsymbol{0} & \boldsymbol{I}_B \end{pmatrix}, \tag{6}$$

and $\boldsymbol{\Sigma}_{T/B}^a = \left(\boldsymbol{\Sigma}_{T/B}^r\right)^{\dagger}$. Finally, the broadening matrices are given in terms of the self-energies as:

$$\begin{cases} \boldsymbol{\Gamma}_T & = i[\boldsymbol{\Sigma}_T^r - \boldsymbol{\Sigma}_T^a] = i\left[i\hbar\gamma \begin{pmatrix} \boldsymbol{I}_T & \boldsymbol{0} \\ \boldsymbol{0} & \boldsymbol{0} \end{pmatrix} - (-i\hbar\gamma)\begin{pmatrix} \boldsymbol{I}_T & \boldsymbol{0} \\ \boldsymbol{0} & \boldsymbol{0} \end{pmatrix}\right] = -2\hbar\gamma \begin{pmatrix} \boldsymbol{I}_T & \boldsymbol{0} \\ \boldsymbol{0} & \boldsymbol{0} \end{pmatrix} \\ \boldsymbol{\Gamma}_B & = i[\boldsymbol{\Sigma}_B^r - \boldsymbol{\Sigma}_B^a] = i\left[i\hbar\gamma \begin{pmatrix} \boldsymbol{0} & \boldsymbol{0} \\ \boldsymbol{0} & \boldsymbol{I}_B \end{pmatrix} - (-i\hbar\gamma)\begin{pmatrix} \boldsymbol{0} & \boldsymbol{0} \\ \boldsymbol{0} & \boldsymbol{I}_B \end{pmatrix}\right] = -2\hbar\gamma \begin{pmatrix} \boldsymbol{0} & \boldsymbol{0} \\ \boldsymbol{0} & \boldsymbol{I}_B \end{pmatrix} \end{cases}. \tag{7}$$

The broadening, $\hbar\gamma$, is chosen to be sufficiently large to obtain a smooth density of states of the top and bottom flakes to mimic their periodic counterparts. The results are tested to be insensitive to this choice (see Supplementary note 5).

For computational efficiency we transform the transmittance probability expression of Supplementary Eq. (3) to the diagonal basis of the dressed Hamiltonian:

$$\boldsymbol{H}_d^{\mathrm{r}} = \boldsymbol{h}_d + \boldsymbol{\Sigma}_B^r + \boldsymbol{\Sigma}_T^r. \tag{8}$$

To this end, we denote by $\boldsymbol{U}$ the transformation matrix that transforms the complex symmetric matrix $\boldsymbol{H}_d^{\mathrm{r}}$ to its diagonal representation $\widetilde{\boldsymbol{H}}_d^r$:

$$\widetilde{\boldsymbol{H}}_d^{\mathrm{r}} = \boldsymbol{U}^{-1}\boldsymbol{H}_d^r\boldsymbol{U} \tag{9}$$

By inserting $\boldsymbol{U}\boldsymbol{U}^{-1}$ or its conjugate transpose between each pair of matrices in Supplementary Eq. (3) and using the cyclic property of the trace operation we obtain:

$$T(E) = \mathrm{Tr}[\boldsymbol{G}_d^r(\boldsymbol{U}\boldsymbol{U}^{-1})\boldsymbol{\Gamma}_B(\boldsymbol{U}\boldsymbol{U}^{-1})^{\dagger}\boldsymbol{G}_d^a(\boldsymbol{U}\boldsymbol{U}^{-1})^{\dagger}\boldsymbol{\Gamma}_T(\boldsymbol{U}\boldsymbol{U}^{-1})] = \mathrm{Tr}\left[\underbrace{\boldsymbol{U}^{-1}\boldsymbol{G}_d^r\boldsymbol{U}}_{\equiv\widetilde{\boldsymbol{G}}_d^r}\underbrace{\boldsymbol{U}^{-1}\boldsymbol{\Gamma}_B(\boldsymbol{U}^{-1})^{\dagger}}_{\equiv\widetilde{\boldsymbol{\Gamma}}_B}\underbrace{\boldsymbol{U}^{\dagger}\boldsymbol{G}_d^a(\boldsymbol{U}^{-1})^{\dagger}}_{\equiv\widetilde{\boldsymbol{G}}_d^a}\underbrace{\boldsymbol{U}^{\dagger}\boldsymbol{\Gamma}_T\boldsymbol{U}}_{\equiv\widetilde{\boldsymbol{\Gamma}}_T}\right] = \mathrm{Tr}\left[\widetilde{\boldsymbol{G}}_d^r\widetilde{\boldsymbol{\Gamma}}_B\widetilde{\boldsymbol{G}}_d^a\widetilde{\boldsymbol{\Gamma}}_T\right]. \tag{10}$$

Note that, in the new basis, the retarded and advanced Green's functions matrices still obey the required relation:

$$\widetilde{\boldsymbol{G}}_d^a = \boldsymbol{U}^{\dagger}\boldsymbol{G}_d^a(\boldsymbol{U}^{-1})^{\dagger} = [\boldsymbol{U}^{-1}(\boldsymbol{G}_d^a)^{\dagger}\boldsymbol{U}]^{\dagger} = [\boldsymbol{U}^{-1}\boldsymbol{G}_d^r\boldsymbol{U}]^{\dagger} = \widetilde{\boldsymbol{G}}_d^{r\,\dagger} \tag{11}$$

and have diagonal representations:

$$\widetilde{\boldsymbol{G}}_d^r = \boldsymbol{U}^{-1}\boldsymbol{G}_d^r\boldsymbol{U} = \boldsymbol{U}^{-1}(E\boldsymbol{I} - \boldsymbol{H}_d^r)^{-1}\boldsymbol{U} = [\boldsymbol{U}^{-1}(E\boldsymbol{I} - \boldsymbol{H}_d^r)\boldsymbol{U}]^{-1} = [E\boldsymbol{U}^{-1}\boldsymbol{I}\boldsymbol{U} - \boldsymbol{U}^{-1}\boldsymbol{H}_d^r\boldsymbol{U}]^{-1} = \left[E\boldsymbol{I} - \widetilde{\boldsymbol{H}}_d^r\right]^{-1}. \quad (12)$$

This allows us, at the expense of a single complex symmetric matrix diagonalization and a single evaluation of $\widetilde{\boldsymbol{\Gamma}}_B$ and $\widetilde{\boldsymbol{\Gamma}}_T$, to evaluate $\widetilde{\boldsymbol{G}}_d^r(E)$ and $\widetilde{\boldsymbol{G}}_d^a(E)$ at any value of E while avoiding matrix inversion. Note also that, since $\boldsymbol{H}_d^{\mathrm{r}}$ is complex symmetric, $\boldsymbol{U}$ is complex orthogonal such that $\boldsymbol{U}^T = \boldsymbol{U}^{-1}$ (or $\boldsymbol{U}^* = (\boldsymbol{U}^{-1})^\dagger$) and $\boldsymbol{U}^\dagger = (\boldsymbol{U}^{-1})^*$ [2]. Hence, we have:

$$\begin{cases} \widetilde{\boldsymbol{G}}_d^r \equiv \boldsymbol{U}^{-1}\boldsymbol{G}_d^r\boldsymbol{U} = \boldsymbol{U}^{\mathrm{T}}\boldsymbol{G}_d^r\boldsymbol{U} \\ \widetilde{\boldsymbol{\Gamma}}_B \equiv \boldsymbol{U}^{-1}\boldsymbol{\Gamma}_B(\boldsymbol{U}^{-1})^\dagger = \boldsymbol{U}^{\mathrm{T}}\boldsymbol{\Gamma}_B\boldsymbol{U}^* \\ \widetilde{\boldsymbol{G}}_d^a \equiv \boldsymbol{U}^\dagger\boldsymbol{G}_d^a(\boldsymbol{U}^{-1})^\dagger = (\boldsymbol{U}^{-1})^*\boldsymbol{G}_d^a\boldsymbol{U}^* \\ \widetilde{\boldsymbol{\Gamma}}_T \equiv \boldsymbol{U}^\dagger\boldsymbol{\Gamma}_T\boldsymbol{U} = (\boldsymbol{U}^{-1})^*\boldsymbol{\Gamma}_T\boldsymbol{U} \end{cases} \quad (13)$$

To evaluate the separate contributions of the edge and bulk sections of the graphene bilayer junction to the total current we classify the various elements of the broadening matrices $\widetilde{\boldsymbol{\Gamma}}_B$ and $\widetilde{\boldsymbol{\Gamma}}_T$ according to their corresponding spatial location. To this end, we write the tight-binding Hamiltonian of the graphene bilayer in the following block form:

$$\boldsymbol{H} = \begin{pmatrix} \boldsymbol{h}_T^e & \boldsymbol{v}_T^{eb} & \boldsymbol{v}_{TB}^{ee} & \boldsymbol{v}_{TB}^{eb} \\ \boldsymbol{v}_T^{be} & \boldsymbol{h}_T^b & \boldsymbol{v}_{TB}^{be} & \boldsymbol{v}_{TB}^{bb} \\ \boldsymbol{v}_{BT}^{ee} & \boldsymbol{v}_{BT}^{eb} & \boldsymbol{h}_B^e & \boldsymbol{v}_B^{eb} \\ \boldsymbol{v}_{BT}^{be} & \boldsymbol{v}_{BT}^{bb} & \boldsymbol{v}_B^{be} & \boldsymbol{h}_B^b \end{pmatrix}. \quad (14)$$

Where $\boldsymbol{h}_{T(B)}^e$ is a block corresponding to all top (bottom) flake sites residing in a narrow region around the edge of the flake, $\boldsymbol{h}_{T(B)}^b$ represents all the complementary top (bottom) flake bulk sites, and the various $\boldsymbol{v}$ blocks are the corresponding inter-block coupling matrices. In this representation the diagonal broadening matrices are written as:

$$\boldsymbol{\Gamma}_T = -2\hbar\gamma\begin{pmatrix} \boldsymbol{I}_T^e & \boldsymbol{0} & \boldsymbol{0} & \boldsymbol{0} \\ \boldsymbol{0} & \boldsymbol{I}_T^b & \boldsymbol{0} & \boldsymbol{0} \\ \boldsymbol{0} & \boldsymbol{0} & \boldsymbol{0} & \boldsymbol{0} \\ \boldsymbol{0} & \boldsymbol{0} & \boldsymbol{0} & \boldsymbol{0} \end{pmatrix} = -2\hbar\gamma\begin{pmatrix} \boldsymbol{I}_T^e & \boldsymbol{0} & \boldsymbol{0} & \boldsymbol{0} \\ \boldsymbol{0} & \boldsymbol{0} & \boldsymbol{0} & \boldsymbol{0} \\ \boldsymbol{0} & \boldsymbol{0} & \boldsymbol{0} & \boldsymbol{0} \\ \boldsymbol{0} & \boldsymbol{0} & \boldsymbol{0} & \boldsymbol{0} \end{pmatrix} - 2\hbar\gamma\begin{pmatrix} \boldsymbol{0} & \boldsymbol{0} & \boldsymbol{0} & \boldsymbol{0} \\ \boldsymbol{0} & \boldsymbol{I}_T^b & \boldsymbol{0} & \boldsymbol{0} \\ \boldsymbol{0} & \boldsymbol{0} & \boldsymbol{0} & \boldsymbol{0} \\ \boldsymbol{0} & \boldsymbol{0} & \boldsymbol{0} & \boldsymbol{0} \end{pmatrix} \equiv \boldsymbol{\Gamma}_T^e + \boldsymbol{\Gamma}_T^b \quad (15)$$

$$\boldsymbol{\Gamma}_B = -2\hbar\gamma\begin{pmatrix} \boldsymbol{0} & \boldsymbol{0} & \boldsymbol{0} & \boldsymbol{0} \\ \boldsymbol{0} & \boldsymbol{0} & \boldsymbol{0} & \boldsymbol{0} \\ \boldsymbol{0} & \boldsymbol{0} & \boldsymbol{I}_B^e & \boldsymbol{0} \\ \boldsymbol{0} & \boldsymbol{0} & \boldsymbol{0} & \boldsymbol{I}_B^b \end{pmatrix} = -2\hbar\gamma\begin{pmatrix} \boldsymbol{0} & \boldsymbol{0} & \boldsymbol{0} & \boldsymbol{0} \\ \boldsymbol{0} & \boldsymbol{0} & \boldsymbol{0} & \boldsymbol{0} \\ \boldsymbol{0} & \boldsymbol{0} & \boldsymbol{I}_B^e & \boldsymbol{0} \\ \boldsymbol{0} & \boldsymbol{0} & \boldsymbol{0} & \boldsymbol{0} \end{pmatrix} - 2\hbar\gamma\begin{pmatrix} \boldsymbol{0} & \boldsymbol{0} & \boldsymbol{0} & \boldsymbol{0} \\ \boldsymbol{0} & \boldsymbol{0} & \boldsymbol{0} & \boldsymbol{0} \\ \boldsymbol{0} & \boldsymbol{0} & \boldsymbol{0} & \boldsymbol{0} \\ \boldsymbol{0} & \boldsymbol{0} & \boldsymbol{0} & \boldsymbol{I}_B^b \end{pmatrix} \equiv \boldsymbol{\Gamma}_B^e + \boldsymbol{\Gamma}_B^b \quad (16)$$

Substituting these definitions into the transmittance probability expression of Supplementary Eq. (10) we obtain:

$$T(E) = \mathrm{Tr}\{\widetilde{\boldsymbol{G}}_d^r[\boldsymbol{U}^{-1}(\boldsymbol{\Gamma}_B^e + \boldsymbol{\Gamma}_B^b)(\boldsymbol{U}^{-1})^\dagger]\widetilde{\boldsymbol{G}}_d^a[\boldsymbol{U}^\dagger(\boldsymbol{\Gamma}_T^e + \boldsymbol{\Gamma}_T^b)\boldsymbol{U}]\} = \mathrm{Tr}[\widetilde{\boldsymbol{G}}_d^r(\widetilde{\boldsymbol{\Gamma}}_B^e + \widetilde{\boldsymbol{\Gamma}}_B^b)\widetilde{\boldsymbol{G}}_d^a(\widetilde{\boldsymbol{\Gamma}}_T^e + \widetilde{\boldsymbol{\Gamma}}_T^b)] =$$

$$\underbrace{\mathrm{Tr}[\widetilde{\boldsymbol{G}}_d^r\widetilde{\boldsymbol{\Gamma}}_B^e\widetilde{\boldsymbol{G}}_d^a\widetilde{\boldsymbol{\Gamma}}_T^e]}_{\equiv T_{e\to e}(E)} + \underbrace{\mathrm{Tr}[\widetilde{\boldsymbol{G}}_d^r\widetilde{\boldsymbol{\Gamma}}_B^e\widetilde{\boldsymbol{G}}_d^a\widetilde{\boldsymbol{\Gamma}}_T^b]}_{\equiv T_{e\to b}(E)} + \underbrace{\mathrm{Tr}[\widetilde{\boldsymbol{G}}_d^r\widetilde{\boldsymbol{\Gamma}}_B^b\widetilde{\boldsymbol{G}}_d^a\widetilde{\boldsymbol{\Gamma}}_T^e]}_{\equiv T_{b\to e}(E)} + \underbrace{\mathrm{Tr}[\widetilde{\boldsymbol{G}}_d^r\widetilde{\boldsymbol{\Gamma}}_B^b\widetilde{\boldsymbol{G}}_d^a\widetilde{\boldsymbol{\Gamma}}_T^b]}_{\equiv T_{b\to b}(E)}, \quad (17)$$

where we defined $\widetilde{\boldsymbol{\Gamma}}_B^{e/b} \equiv \boldsymbol{U}^{-1}\boldsymbol{\Gamma}_B^{e/b}(\boldsymbol{U}^{-1})^\dagger = \boldsymbol{U}^{\mathrm{T}}\boldsymbol{\Gamma}_B^{e/b}\boldsymbol{U}^*$ and $\widetilde{\boldsymbol{\Gamma}}_T^{e/b} \equiv \boldsymbol{U}^\dagger\boldsymbol{\Gamma}_T^{e/b}\boldsymbol{U}$.

The various terms contributing to the transmittance probability in Supplementary Eq. (17) can be interpreted as follows: $T_{e\to e}$ describes the probability of electrons entering the junction at the edge region of the bottom layer to exit at the edge region of the top layer; $T_{e\to b}$ describes the probability of electrons entering the junction at the edge region of the bottom layer to exit at the bulk region of the top layer; $T_{b\to e}$ describes the probability of electrons entering the junction at the bulk region of the bottom layer to exit at the edge region of the top layer; and $T_{b\to b}$ describes the probability of electrons entering the junction at the bulk region of the bottom layer to exit at the bulk region of the top layer. Finally, the different current contributions are obtained using the Landauer expression (Supplementary Eq. (1)), where the edge current is evaluated using $T_{e\to e}(E)$,the bulk current is evaluated using $T_{b\to b}(E)$, and the cross contributions are obtained by using $T_{e\to b}(E)$ and $T_{b\to e}(E)$.

It is important to note that the four transmittance probability contributions presented above ($T_{e\to e}$, $T_{e\to b}$, $T_{b\to e}$, and $T_{b\to b}$) only specify where electrons enter and exit the bilayer structure regardless of the specific route that they take when crossing the interface itself. This latter information is embedded in the Green's functions appearing in the trace formula (Supplementary Eq. (3)) and cannot be rigorously separated into bulk and edge contributions when there is coupling between the two regions that leads to delocalization of the wave functions over the entire flake surface.

We comment that the experimental values are analyzed under the assumption that the edge and the bulk contributions can be treated as parallel resistors. In practice, this means that the coupling between the edge and bulk regions can be neglected or that the wave packet of the transmitting electrons is sufficiently localized to pass through a specific surface region. These assumptions become valid with increasing junction lateral dimensions.

5. Validity Test for the Value of the Broadening Factors

Within our approximate treatment of the electronic transport problem the coupling of the modeled interface to implicit particle reservoirs is done by introducing broadening factors, $\hbar\gamma$, which mimic the imaginary part of the implicit bath self-energy within the wide-band approximation. This introduces a finite lifetime to the atomic states of the graphene flakes to which the broadening factors are applied. In energy space this translates to broadening of the δ-function eigenstates of the isolated flake into Lorentzian functions of width $\hbar\gamma$. To avoid artefacts resulting from the discreteness of the spectrum of the finite model flake, the value of the broadening factors should be sufficiently large to result in a continuous density of states (DOS). Care should be taken also not to use too large broadenings to allow for the characteristic electronic structure of the flakes to be manifested in the calculation.

To validate that our choice of broadening factors fulfils these requirements we plot the density of states of one of the flakes constructing the interface while assigning each eigenvalue, ε_n, a Lorentzian function of width $\hbar\gamma$ such that the total DOS is calculated as follows:

$$DOS(\varepsilon) = \frac{1}{2\pi}\sum_n \frac{\hbar\gamma}{(\varepsilon-\varepsilon_n)^2+(\hbar\gamma/2)^2}. \qquad (18)$$

In Supplementary Figure (4) we illustrate the DOS of the two zigzag hexagonal graphene flakes considered herein (side lengths of (a) 6.2 and (b) 7.6 nm), calculated using broadening factors of $\hbar\gamma = 0.01$ (blue), 0.05 (red), and 0.1 (orange) eV. For both systems, at a value of $\hbar\gamma = 0.05$ eV, the discrete nature of the flake levels is sufficiently smeared to provide a relatively smooth DOS curve without washing-out the unique features of the electronic structure of the system. Since all other systems considered in this study are of similar or larger dimensions and nature, we adopted this value of the broadening factor throughout.

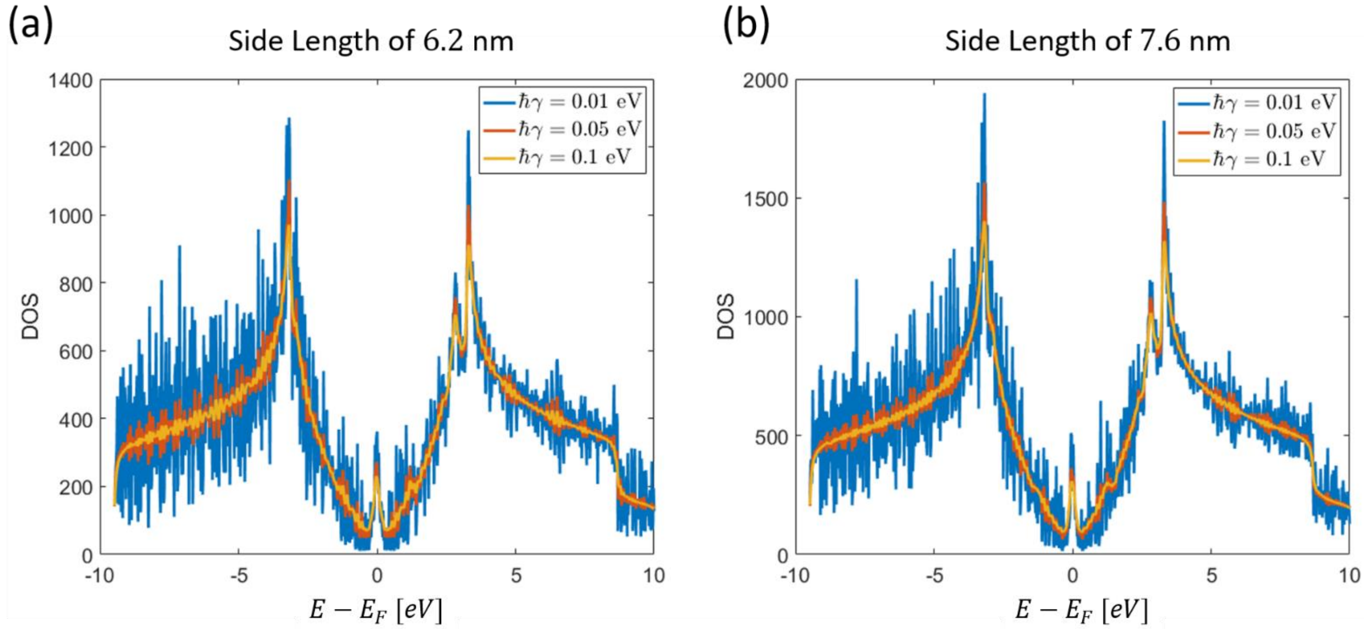

Supplementary Figure 4: Illustration of the broadened DOS. **(a)** 6.2 nm and **(b)** 7.6 nm zigzag hexagonal graphene flakes calculated using Lorentzian broadening with width factors of $\hbar\gamma = 0.01$ (blue), 0.05 (red), and 0.1 (orange) eV.

To further validate our choice of broadening factors we plot in Supplementary Fig. 4 the transmittance probability, $T(E)$, of the two-layer zigzag hexagonal graphene junction with flake side-length of 6.2 nm and a misfit angle of $15°$ for several values of the broadening factors. We note that the broadening in these calculations are applied directly to the interfacing flakes, hence the transmittance curves are relatively broad (see Supplementary Information section 6 for a discussion of the consequences of applying the broadening directly to the coupled flakes). While for the smallest broadening factor depicted herein ($\hbar\gamma = 0.025$ eV) the $T(E)$ curve differs from that obtained with the value used in the main text ($\hbar\gamma = 0.05$ eV), increasing the broadening factor to $\hbar\gamma = 0.075$ and 0.1 eV has a small effect on the transmittance curve thus indicating that our calculations are performed within the stability range of this parameter.

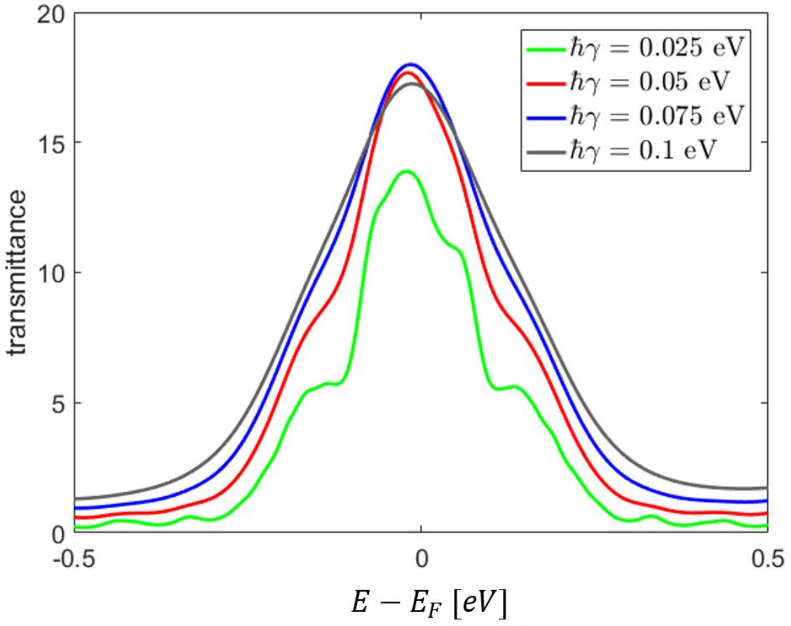

Supplementary Figure 5: Transmittance probability of zigzag terminated bilayer graphene. Calculated for the two-layer zigzag hexagonal graphene junction with flake side-length of 6.2 nm and a misfit angle of 15° for several values of the broadening factors: $\hbar\gamma = 0.025$ eV (green line), 0.05 eV (red line), 0.075 eV (blue line), and 0.1 eV (gray line).

6. Convergence Test with respect to the Number of Modeled Layers

The results presented in the main text were obtained using a bilayer model system with broadening factors applied directly to the atoms of the contacting layers. This allowed us to perform calculations on large-scale junctions with reasonable computational burden. However, contacting the interfacing layers directly to the implicit leads has the effect of exaggerated broadening of the transmittance probability curves. Therefore, to confirm the validity of our qualitative conclusions, we repeated some of the calculations using a four-layer model system.

The Hamiltonian of the device can be written in block form as follows:

$$\boldsymbol{H}_d = \begin{pmatrix} \boldsymbol{H}_T & \boldsymbol{H}_{TC} & \boldsymbol{H}_{TB} \\ \boldsymbol{H}_{CT} & \boldsymbol{H}_C & \boldsymbol{H}_{CB} \\ \boldsymbol{H}_{BT} & \boldsymbol{H}_{BC} & \boldsymbol{H}_B \end{pmatrix}, \tag{19}$$

where $\boldsymbol{H}_T$, $\boldsymbol{H}_C$ and $\boldsymbol{H}_B$ are the Hamiltonian blocks of the top graphene flake, central two flakes, and bottom flake, respectively, and the broadening factors are applied only to the top and the bottom flake atoms in the following manner:

$$\boldsymbol{\Sigma}_T^r = i\hbar\gamma \begin{pmatrix} \boldsymbol{I}_T & \boldsymbol{0} & \boldsymbol{0} \\ \boldsymbol{0} & \boldsymbol{0} & \boldsymbol{0} \\ \boldsymbol{0} & \boldsymbol{0} & \boldsymbol{0} \end{pmatrix}, \ \boldsymbol{\Sigma}_B^r = i\hbar\gamma \begin{pmatrix} \boldsymbol{0} & \boldsymbol{0} & \boldsymbol{0} \\ \boldsymbol{0} & \boldsymbol{0} & \boldsymbol{0} \\ \boldsymbol{0} & \boldsymbol{0} & \boldsymbol{I}_B \end{pmatrix}. \tag{20}$$

Supplementary Figures 6-8 compare the total transmittance probability curves, as well as their division into bulk and edge contributions, for the 6.53 nm side-length armchair hexagonal (Supplementary Fig. 6), 6.15 nm side-length zigzag hexagonal (Supplementary Fig. 7), and 10 nm diameter circular (Supplementary Fig. 8) flakes with two (panels (a)) and four (panels (b)) layers. While the four-layer model system provides narrower transmittance features than the two-layer model, where the contacting layers are directly coupled to the corresponding implicit lead, the qualitative nature of the results remains the same. Namely, for both zigzag hexagonal and circular junctions the edge-to-edge transport contribution dominates, whereas for the armchair hexagonal contact bulk-to-bulk transmittance governs the transport properties of the junction. Furthermore, the relative importance of all other contributions remains intact thus justifying our usage of the two-layer model systems to perform the demanding large-scale interlayer transport calculations.

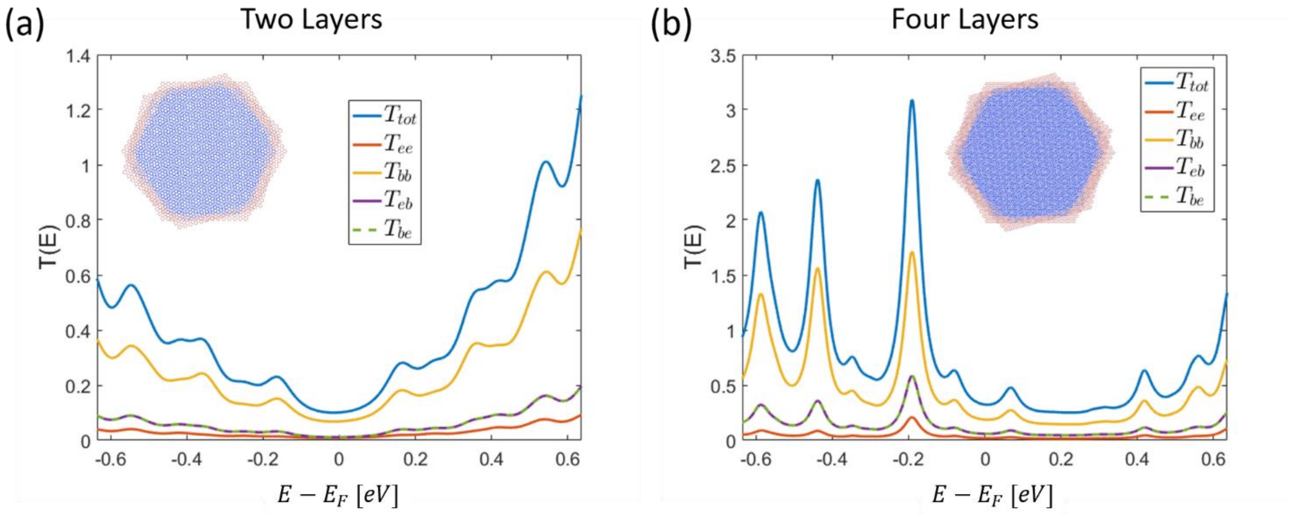

Supplementary Figure 6: Calculated transmittance probability for armchair terminated bilayer graphene. Total transmittance probability (blue line) and its edge and bulk contributions (red, orange, purple, and dashed green lines) calculated for **(a)** two-layer and **(b)** four-layer armchair hexagonal graphene junctions of side length of 6.53 nm and a misfit angle of 15° (see insets).

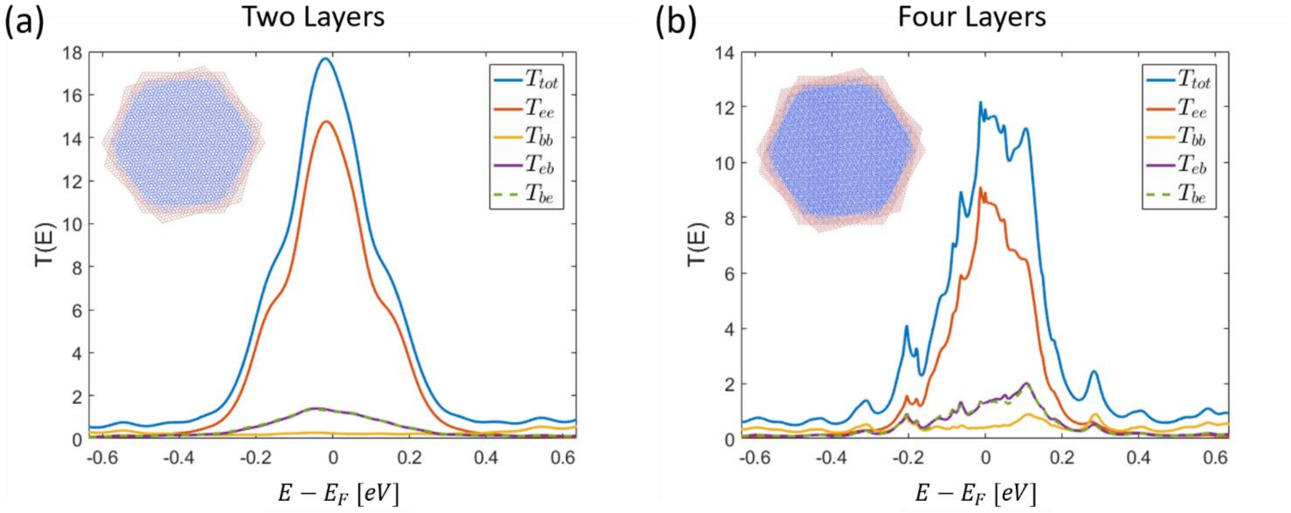

Supplementary Figure 7: Calculated transmittance probability for zigzag terminated bilayer graphene. Total transmittance probability (blue line) and its edge and bulk contributions (red, orange, purple, and dashed green lines) calculated for **(a)** two-layer and **(b)** four-layer zigzag hexagonal graphene junctions of side length of 6.15 nm and a misfit angle of 15° (see insets).

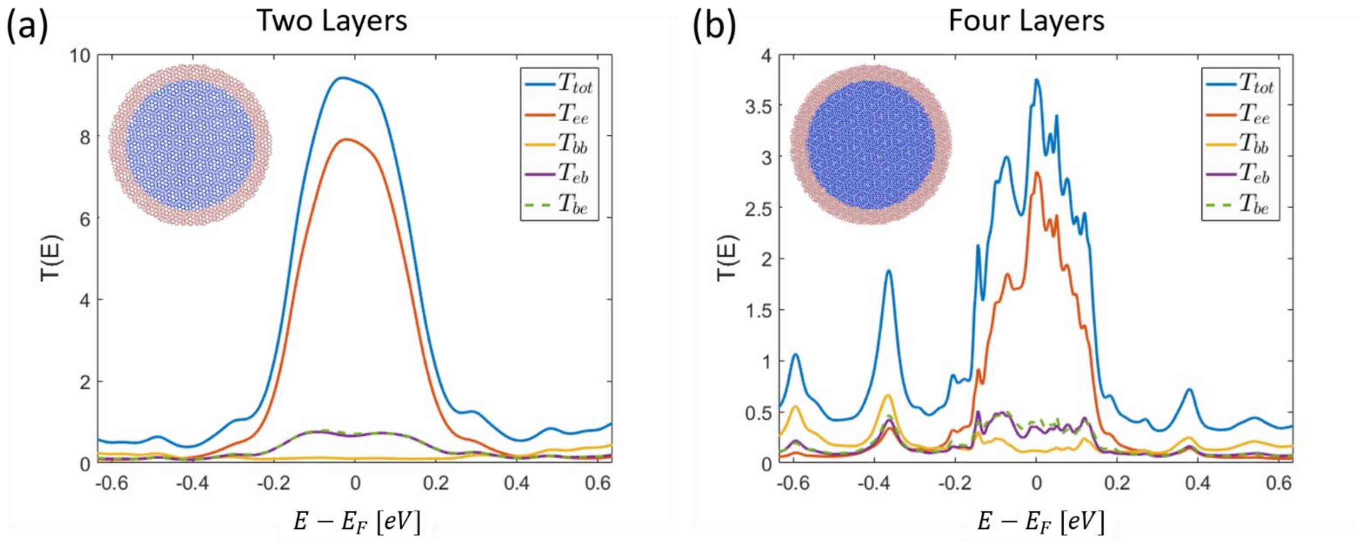

Supplementary Figure 8: Calculated transmittance probability for circular bilayer graphene structures. Total transmittance probability (blue line) and its edge and bulk contributions (red, orange, purple, and dashed green lines) calculated for **(a)** two-layer and **(b)** four-layer 10 nm diameter circular graphene junctions and a misfit angle of 15° (see insets).

7. Illustration of the Wave Functions of Eight Eigenstates Within the Fermi Window of Circular Junctions

To obtain the molecular orbital plots of the edge states presented in Fig. 3a-c of the main text we diagonalized the tight-binding Hamiltonian[6] of the relevant junction models and plotted the absolute squared molecular orbital expansion coefficients over the different atomic sites. In Fig. 3d of the main text we presented the angularly averaged molecular orbital weights as a function of distance from the flake edge averaged over eight molecular orbitals residing within the Fermi window. For completeness, we present in Supplementary Fig. 9 the individual eight molecular orbital absolute squared weights of the 20 nm circular bilayer system all showing pronounced edge character. The corresponding angularly averaged molecular orbitals weights as a function of distance from the flake edge are presented to the right of each molecular orbital for the 10 nm (blue circles), 15 nm (red circles) and 20 nm (grey circles) junctions.

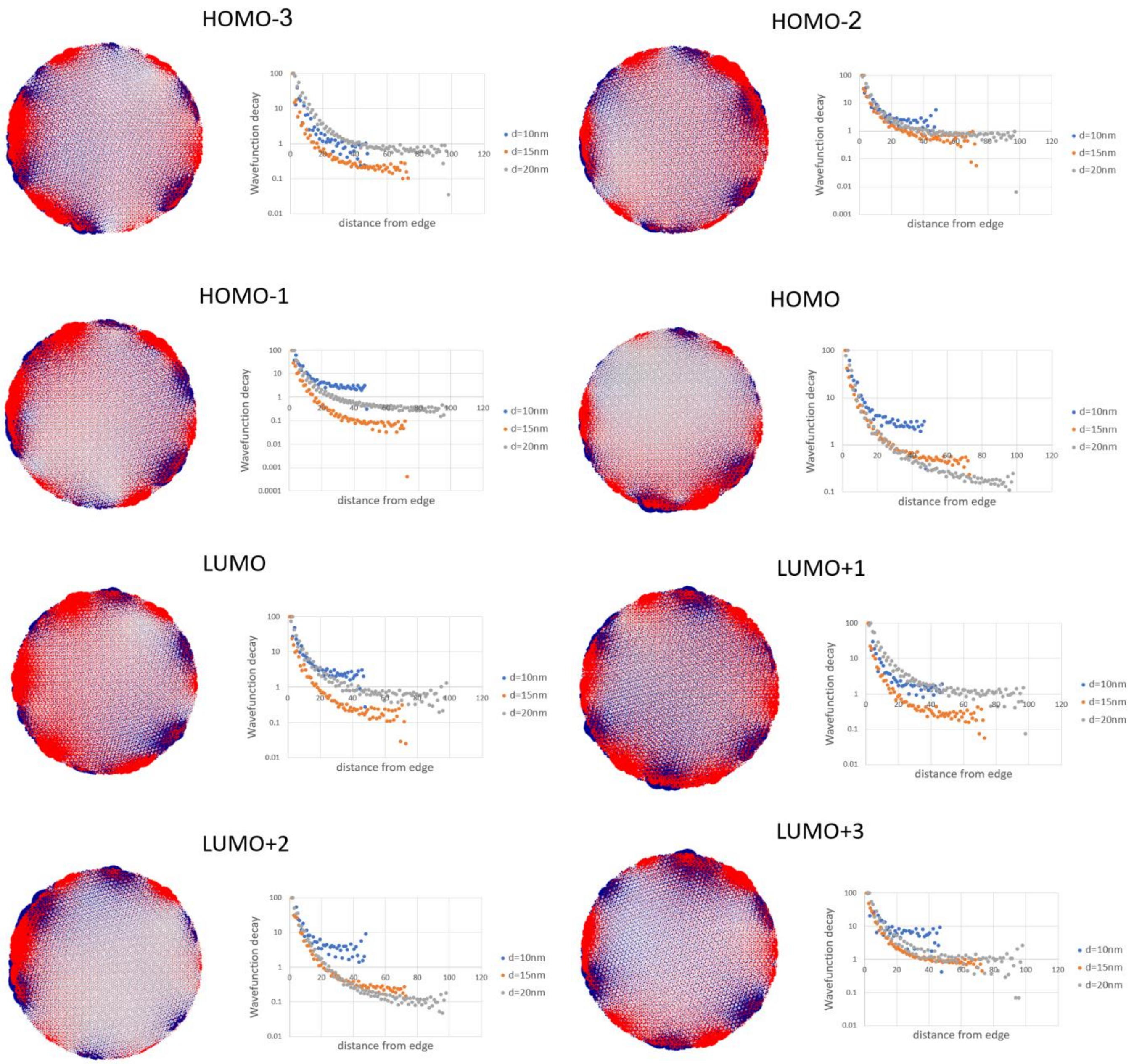

Supplementary Figure 9: Illustrations of the eight molecular orbitals considered in the main text for a bilayer 20 nm diameter circular junction with a misfit angle of 15°. For clarity of the presentation, the weights over the lower and upper flakes are represented in blue and red colors, respectively. Angularly averaged molecular orbital weights as function of distance from the flake edge (calculated by summing absolute squared expansion coefficients over concentric rings) for bilayer junctions of diameters of 10 nm (blue circles), 15 nm (red circles) and 20 nm (grey circles) and a misfit angle of 15° are presented to the right of the corresponding molecular orbital illustration.

References

1. Koren, E., Lortscher, E., Rawlings, C., Knoll, A. W. & Duerig, U. Adhesion and friction in mesoscopic graphite contacts. *Science* **348**, 679–683 (2015).
2. Dietzel, D., Feldmann, M., Schwarz, U., Fuchs, H. & Schirmeisen, A. Scaling Laws of Structural Lubricity. *Phys. Rev. Lett.* **111**, (2013).
3. Lee, C. *et al.* Frictional Characteristics of Atomically Thin Sheets. *Science* **328**, 76–80 (2010).
4. Koren, E. *et al.* Coherent commensurate electronic states at the interface between misoriented graphene layers. *Nat. Nanotechnol.* **11**, 752–757 (2016).
5. Cuevas, J. C. & Scheer, E. *Molecular Electronics: An Introduction to Theory and Experiment*. (World Scientific Publishing Company, 2010).
6. Habib, K. M. M., Sylvia, S. S., Ge, S., Neupane, M. & Lake, R. K. The coherent interlayer resistance of a single, rotated interface between two stacks of AB graphite. *Appl. Phys. Lett.* **103**, 243114 (2013).
